# Automated analysis of knee joint alignment using detailed angular values in long leg radiographs based on deep learning

**DOI:** 10.1038/s41598-024-57887-1

**Published:** 2024-03-27

**Authors:** Hong Seon Lee, Sangchul Hwang, Sung-Hwan Kim, Nam Bum Joon, Hyeongmin Kim, Yeong Sang Hong, Sungjun Kim

**Affiliations:** 1 Department of Radiology, Gangnam Severance Hospital, Yonsei University College of Medicine, 211, Eonju-ro, Gangnam-gu, Seoul, Republic of Korea; 2 Research Institute of Radiological Science, Center for Clinical Imaging Data Science, Yonsei University College of Medicine, Seoul, Republic of Korea; 3 Department of Orthopedic Surgery, Gangnam Severance Hospital, Yonsei University College of Medicine, Seoul, Republic of Korea

**Keywords:** Knee joint alignment, Radiograph, Deep learning, Osteoarthritis, Musculoskeletal system, Skeleton

## Abstract

Malalignment in the lower limb structure occurs due to various causes. Accurately evaluating limb alignment in situations where malalignment needs correction is necessary. To create an automated support system to evaluate lower limb alignment by quantifying mechanical tibiofemoral angle (mTFA), mechanical lateral distal femoral angle (mLDFA), medial proximal tibial angle (MPTA), and joint line convergence angle (JLCA) on full-length weight-bearing radiographs of both lower extremities. In this retrospective study, we analysed 404 radiographs from one hospital for algorithm development and testing and 30 radiographs from another hospital for external validation. The performance of segmentation algorithm was compared to that of manual segmentation using the dice similarity coefficient (DSC). The agreement of alignment parameters was assessed using the intraclass correlation coefficient (ICC) for internal and external validation. The time taken to load the data and measure the four alignment parameters was recorded. The segmentation algorithm demonstrated excellent agreement with human-annotated segmentation for all anatomical regions (average similarity: 89–97%). Internal validation yielded good to very good agreement for all the alignment parameters (ICC ranges: 0.7213–0.9865). Interobserver correlations between manual and automatic measurements in external validation were good to very good (ICC scores: 0.7126–0.9695). The computer-aided measurement was 3.44 times faster than was the manual measurement. Our deep learning-based automated measurement algorithm accurately quantified lower limb alignment from radiographs and was faster than manual measurement.

## Introduction

Malalignment of lower limb structure occurs due to congenital, developmental, or post-traumatic causes, leading to knee joint malalignment, causing joint degeneration, abnormal gait, pain, and asymmetric overloading of articular compartments^1^. Tibiofemoral malalignment is considered a risk factor for osteoarthritis (OA), with genu varum and genu valgum increasing the risk of medial and lateral OA progression, respectively. The severity of malalignment is directly related to knee joint function deterioration^2,3^.

Accurate evaluation of limb alignment is necessary for situations where malalignment needs correction, such as limb realignment surgery or joint replacement surgery^4^. Full-length weight-bearing radiographs of the lower extremities in an upright posture are commonly used in clinical settings to evaluate lower limb alignment, joint orientation, and leg length discrepancy^5^. During imaging, the patient stands upright with bare feet together, fully extended knees, and forward-facing patellae to prevent rotation of the lower limbs.

Whole limb alignment is evaluated based on the mechanical tibiofemoral angle (mTFA), mechanical lateral distal femoral angle (mLDFA), medial proximal tibial angle (MPTA), and joint line convergence angle (JLCA). Accurately measuring these parameters is crucial to identify the main source of deformity. Micicoi et al. reported a physiologic value of 85.8° for mLDFA and 85.6° for MPTA, indicating a 4° valgus and 4° varus of femoral and tibial bone morphology, respectively^6^. In patients with OA, varus deformity (hip-knee-ankle angle < 177°) is caused by distal femoral wear (mLDFA = 89°), tibial varus obliquity (MPTA = 87°), and lateral joint line opening (JLCA = 3°)^7^. However, compensating for any measurement abnormalities can achieve a balanced limb position. Therefore, measuring each parameter is vital for comprehending alignment abnormalities and identifying their primary cause^7^. However, this may be a laborious and time-consuming task for radiologists.

**Figure 1:**
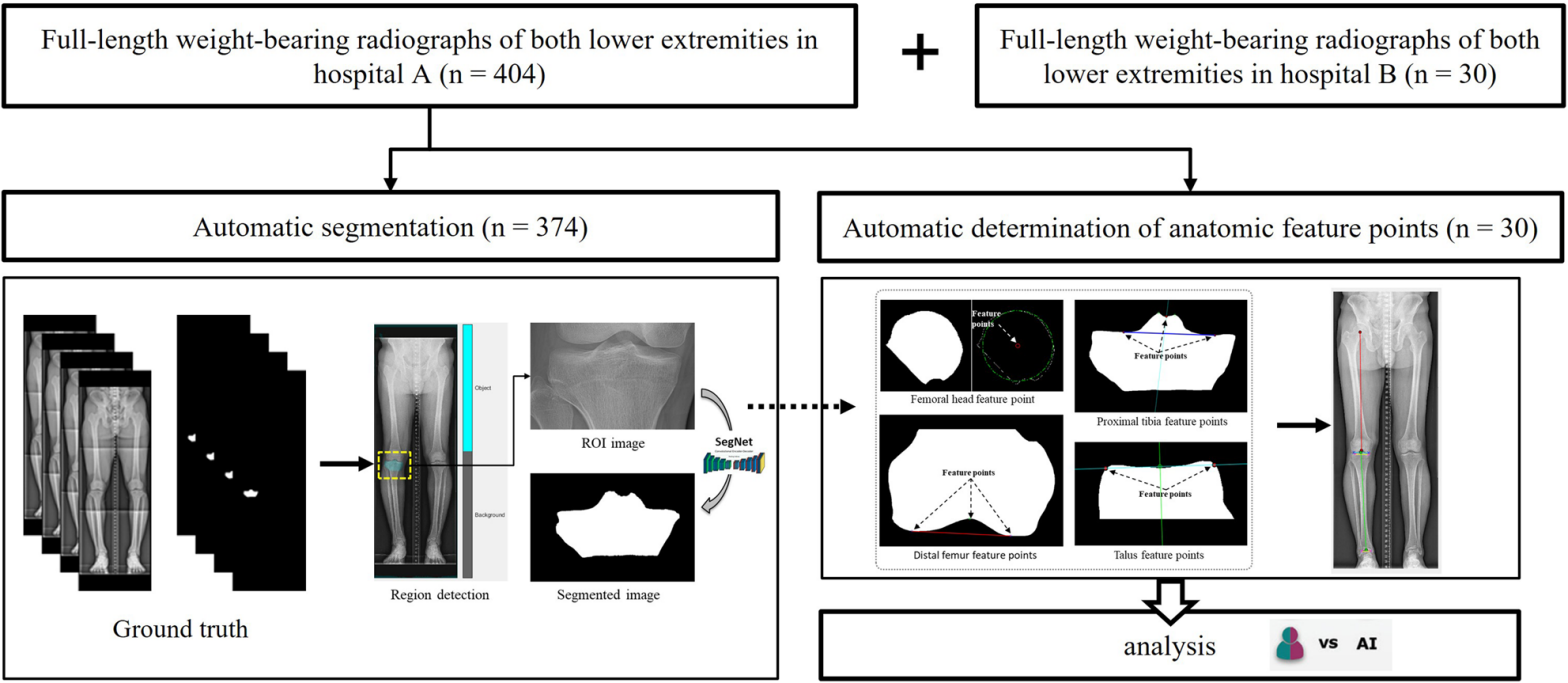
Overview of patient enrollment, algorithm development, and analysis.

Therefore, there is a clinical need for a standardised and reproducible automatic analysis tool that measures lower limb alignment using full-length weight-bearing radiographs^8,9^. Moreover, developing a technical framework based on artificial intelligence applicable in clinical settings is potentially feasible^9^. Our objective was to create, train, and validate an automated support system to evaluate lower limb alignment by quantifying mTFA, mLDFA, MPTA, and JLCA on full-length weight-bearing radiographs of both lower extremities (Fig. 1).

## Materials and methods

### Study participants and radiograph data

**Figure 2:**
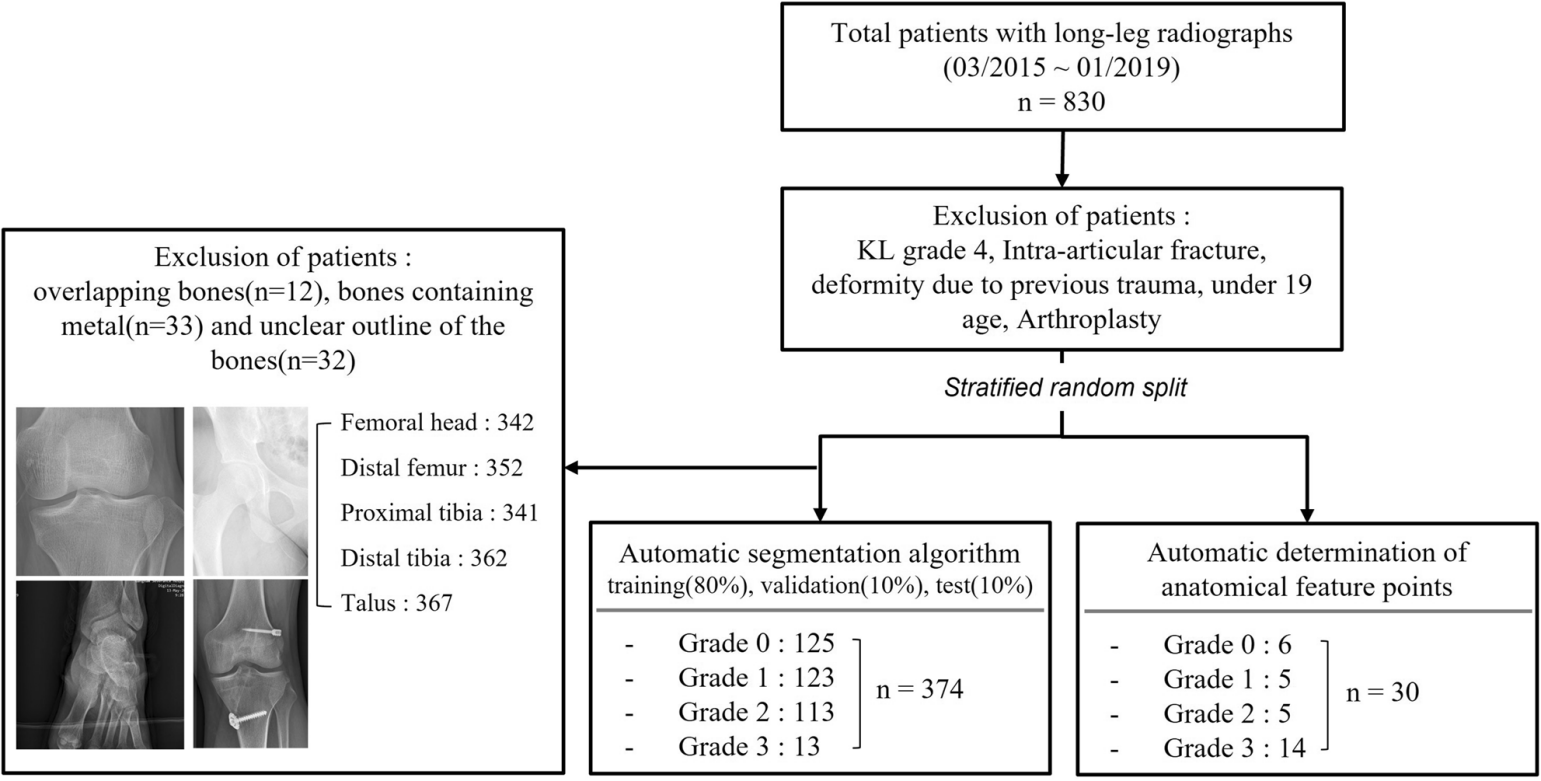
Patient flowchart for algorithm development and clinical verification.

This retrospective study received approval from the institutional review boards of a tertiary hospital (A) (Yonsei University Gangnam Severance Hospital, Institutional Review Board, No 3-2020-0127) and a military hospital (B) (Armed Forces Capital Hospital, Institutional Review Board, 2023-02-002), and informed consent was waived because the data used in this retrospective study were fully de-identified to protect patient confidentiality. All methods were performed in accordance with the ethical standards of Helsinki Declaration. A total of 404 full-length weight-bearing radiographs of both lower extremities from 404 patients (mean age: 44.3 years, 188 men, 186 women) from hospital A were used to develop and test the algorithm. An external test set of 30 consecutive radiographs from 30 men (mean age: 30.2 years) from hospital B was included. The patients underwent long-leg radiography at the two institutions between March 2015 to January 2019 and between August 2022 and September 2022, respectively. Patients from hospital A with K-L grade 4, intra-articular fracture, deformity due to previous trauma, and knee arthroplasty, and those < 19 years were excluded (n = 426) (Fig. 2). The long-leg radiographs were obtained using two imaging acquisition systems and covered the whole lower limbs from the hips to the ankles under single anteroposterior exposure. Philips DigitalDiagnost (Philips, Best, The Netherlands) and Carestream DRX-Evolution (Carestream Health, Rochester, NY, USA) were used in hospitals A and B, respectively.

Next, 30 radiographs out of the 404 were used for clinical verification of the algorithm's anatomical feature points, chosen through stratified random splitting based on the K-L grade. The remaining 374 radiographs were used to develop and validate the automatic segmentation algorithm. Cases with overlapping bones (n = 12), bones containing metal (n = 33), and unclear bone outline (n = 32) were excluded to ensure methodological consistency^10^. For the algorithm’s development, 342 radiographs for the femoral head, 352 for the distal femur, 341 for the proximal tibia, 362 for the distal tibia, and 367 for the talus were used. The collected radiographs were divided into the training set (80%), validation set (10%), and test set (10%) (Fig. 2).

### Manual segmentation

The femoral heads, knee joints, and ankle joints were manually segmented using Adobe Photoshop CC 2018 (Adobe Systems Inc., San Jose, CA, USA) to create masks, which served as the reference for comparison. A radiology technician, supervised by an experienced radiologist, labeled the masks.

### Manual reference measurements

**Figure 3:**
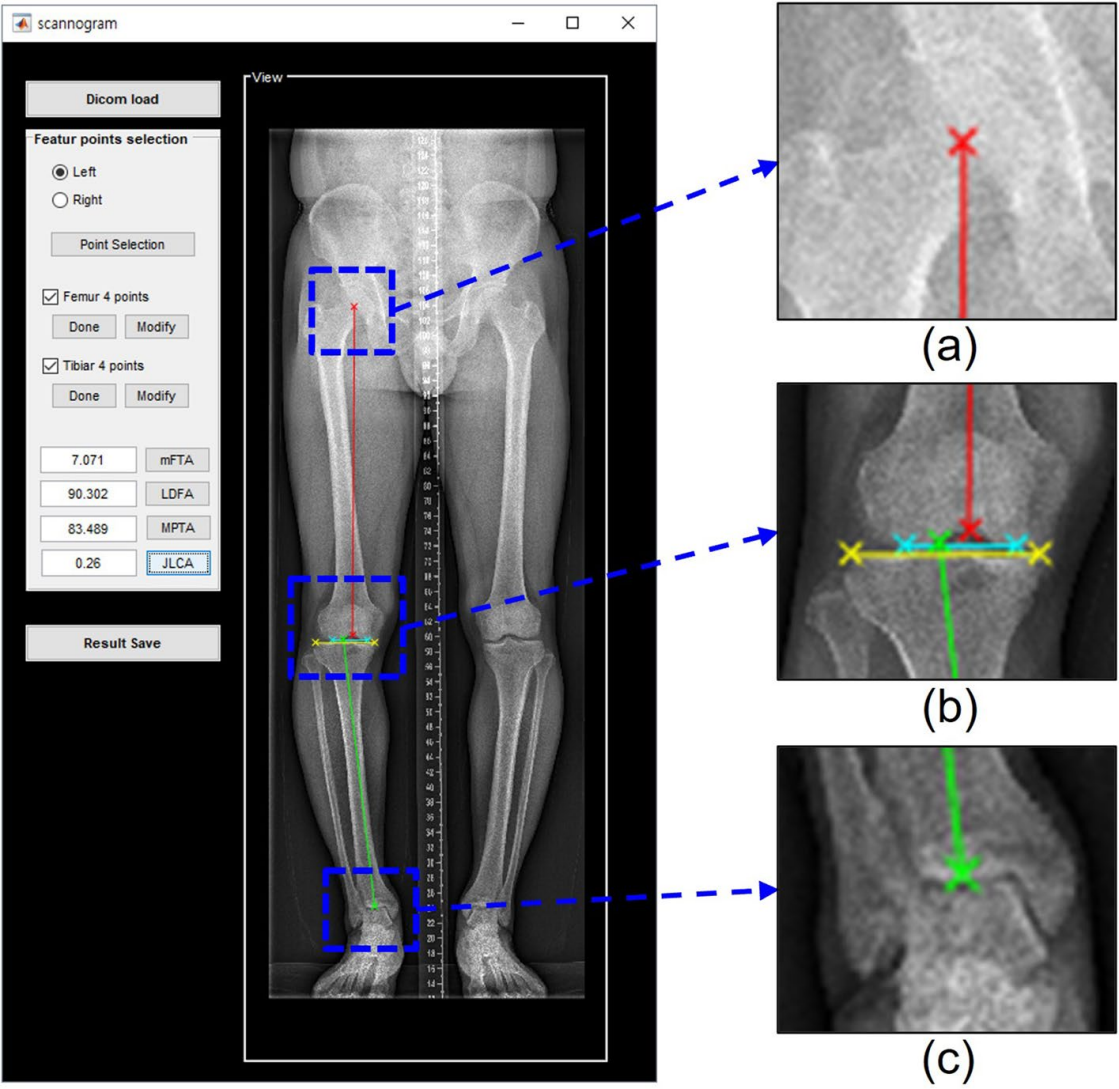
Alignment parameter measurement tool by manually selecting 8 feature points and 4 lines: (**a**) Femoral head centre, (**b**) centre of femoral intercondylar notch, centre of the tibial spines, two most distal points of medial and lateral femoral condyles, and two most proximal points of medial and lateral tibial plateaus and (**c**) mid-malleolar point.

Lower limb alignment was evaluated based on the following anatomic feature points (Fig. 3): (1) the centre of the femoral head, (2) the centre of the femoral intercondylar notch, (3) centres of the medial and lateral tibial spines, (4) two most distal points of the medial and lateral femoral condyles, (5) two most proximal points of the medial and lateral tibial plateaus, and (6) mid-malleolar point (centre of the ankle).

The mechanical axis of the femur was defined as a line drawn from the centre of the femoral head to the centre of the femoral intercondylar notch. The mechanical and anatomical axes of the tibia were defined as the line connecting the centre of the tibial spines and the centre of the ankle. The distal femoral articular axis was defined by the line connecting the most distal points of the medial and lateral femoral condyles. The proximal tibial articular axis was defined as the line connecting the two most proximal points of the tibial plateaus. Four alignment parameters (mTFA, mLDFA, MPTA, and JLCA) were measured using the aforementioned eight feature points and four lines.

We developed a tool for measuring alignment parameters using MATLAB’s Graphical User Interface Development Environment (GUIDE) to create a Graphical User Interface (GUI) in MATLAB. This tool allows the designation of landmarks for angle measurement and calculates the angles using these points (Fig. 3). To assess the intraobserver and interobserver agreement of the measured values between the readers and algorithm, an orthopedic fellow measured the angles of the clinical verification data set (n = 30) twice, with a 2-week interval between the measurement sessions. Another radiology fellow measured the angles once. Regarding the test from the external institution, a fellowship-trained radiologist measured the angles twice. The time taken to load the data and measure the four alignment parameters using the tool was recorded.

### Automated segmentation algorithm

Representative models of Semantic Segmentation include FCN (Fully Convolutional networks), U-Net, and SegNet. FCN needs to learn deconvolution when upsampling, so it needs weight parameters for learning, but in SegNet, this process is omitted, so the learning parameters are reduced. U-Net skip combines during the decoding process, but U-Net transfers the entire feature map information of the same layer from the encoder to the decoder and concats it. Therefore, it is heavier than SegNet, which only selects and uses some features of Max pooling indices.

For this reason, in this study, the outline of each bone was automatically segmented using SegNet. The SegNet architecture consists of a down sampling (encoding) path and a corresponding upsampling (decoding) path, followed by a final pixel-wise classification layer. In the encoder path, there are 13 convolutional layers that match the first 13 convolutional layers in the VGG16 network. Each encoder layer has a corresponding decoder layer; therefore, the decoder network also has 13 convolutional layers. The output of the final decoder layer is fed into a multi-class softmax classifier to produce class probabilities for each pixel independently^11^.

**Figure 4:**
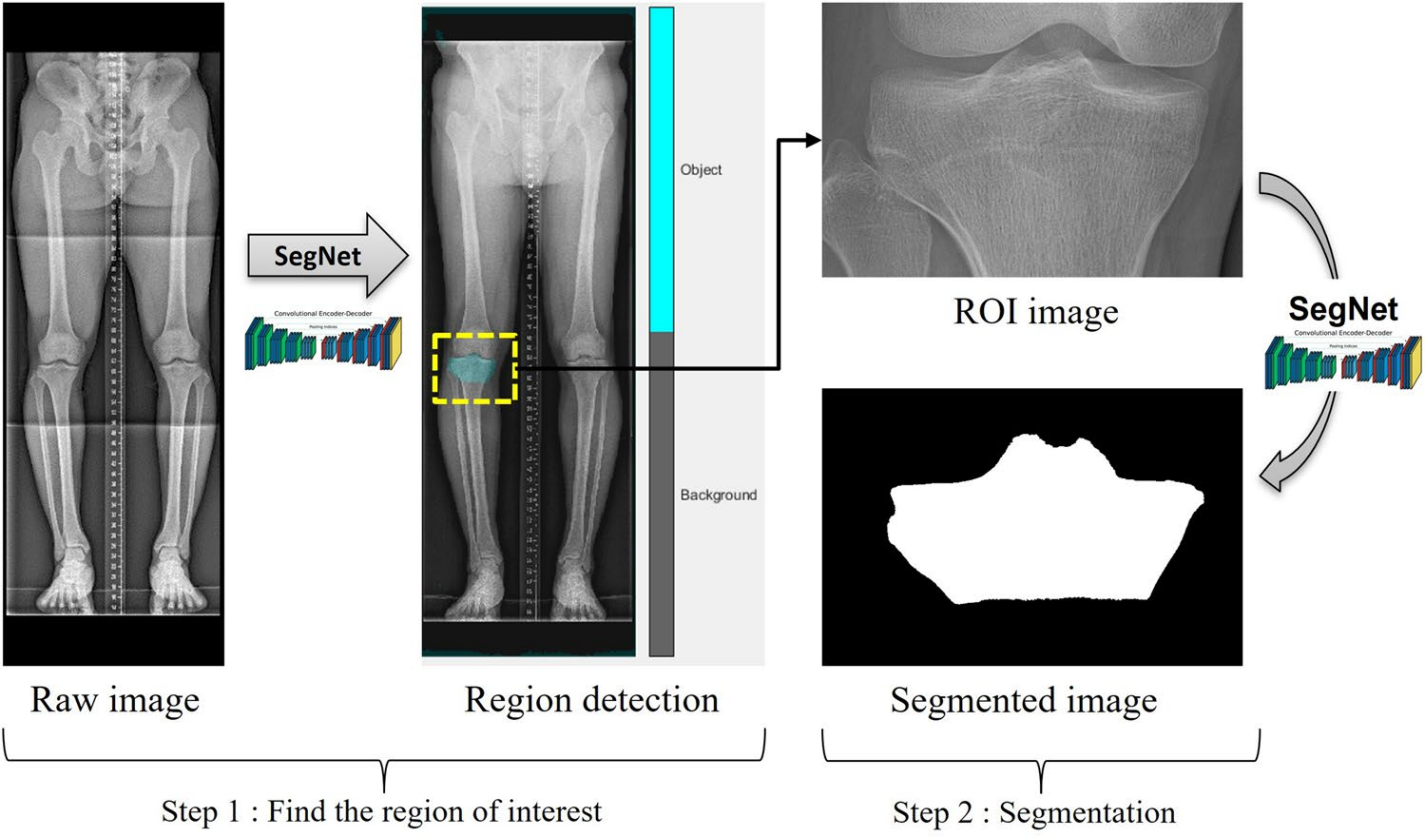
Flowchart of the automatic segmentation algorithm. The first step was performed on raw images. The second step was performed based on the region of interest (ROI) image created by cropping the raw image.

To automatically segment the contours of each bone, we implemented a two-step segmentation algorithm (Fig. 4). In the initial step, we identified the region of interest containing the target bone, and subsequently, in the second step, we delineated the boundaries of the target bones within the identified image region. During the first step, the images were resized to 311 × 932 pixels, and the intensities were scaled to the range [0,1]. In the subsequent step, the images were resized to different pixel dimensions based on the size of each bone (Femoral head: 470 × 470, Distal femur: 740 × 540, Proximal tibia: 720 × 470, Distal tibia: 470 × 430, Talus: 370 × 220), and intensities were scaled to the range [0,1]. We used SGD (Stochastic Gradient Descent) Momentum as the solver to train the deep learning network. The maximum number of Epochs to train the SegNet model was set to 120, and a mini-batch with 4 observations was used for each iteration. And the momentum value was set to 0.9 and the learning rate to 1 × e^−2^. The SegNet model was trained using the training and validation data and implemented with MATLAB R2018b on a GeForce GTX 1080Ti graphics processing unit.

### Automatic determination of anatomic feature points

**Figure 5:**
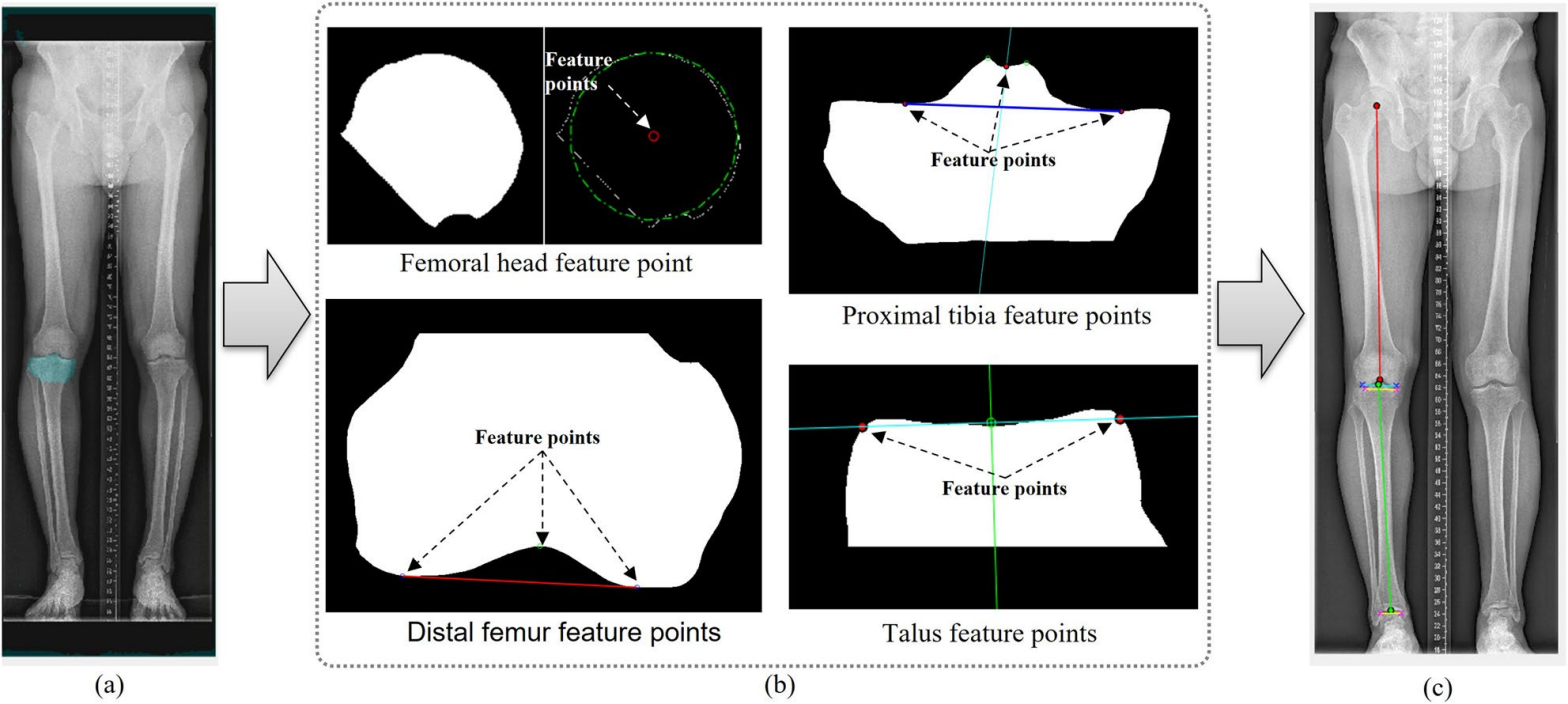
Flowchart of automatic determination algorithm of anatomic feature points. (**a**) Segmented images. (**b**) Anatomic feature points automatically determined based on segmented images. (**c**) The mechanical axes for the lower limb alignment.

The mechanical axes for lower limb alignment were automatically determined based on the segmentation masks (Fig. 5). The computer-aided automatic measurement times from image data loading to determining the four alignment parameters were recorded.

#### The femoral head anatomic feature point

A circle was fit to the segmentation outline of the femoral head to determine its centre.

#### The distal femur anatomic feature point and the distal femur surface line

The region comprising the distal femur surface line and the centroid of the segmentation outline was identified as the distal femur anatomic feature point. The distal femur surface line was determined by minimisig the distance between the bottom line of the bounding box and the segmentation outline, resulting in two points. The highest point within the defined area, encompassing the outline, was designated as the distal femur anatomic feature point.

#### The proximal tibia anatomic feature points

Two peaks were detected from the segmentation outline, and the midpoint between these two points was extracted to determine the proximal tibia anatomic feature points. Next, an orthogonal line was created by connecting the two points and the midpoint, and the position along the segmented outline where the distance between the orthogonal line and the outline was minimised was defined as the proximal tibia anatomic feature point.

#### The proximal tibia surface line

The convex hull^12,13^ and bounding box of the segmentation outline were calculated. To determine the feature points, candidate points were identified by selecting points above the centroid of the segmentation outline within the region defined by the convex hull. Next, the proximal tibia surface line was defined by identifying the two points closest to the upper corner points of the bounding box from the candidate points.

#### Distal tibia anatomic feature points

Two talus feature points were defined by applying the same method of defining the proximal tibia surface line. Next, an orthogonal line was constructed by connecting the midpoint of the two talus feature points, and the position where the distance between the orthogonal line and the segmented outline of the distal tibia was minimum was defined as the distal tibia anatomic feature point.

### Statistical analysis

We implemented global accuracy, mean accuracy, mean intersection over union (IoU), weighted IoU, and the dice similarity coefficient (DSC) to evaluate the segmentation algorithm’s performance, which compares the similarity of the automated segmentation mask with the human-annotated segmentation mask. As a representative measurement, we considered a DSC ≥ 0.7 as indicative of excellent agreement between two segmented regions, following previous studies^14,15^.

We confirmed normality in each group for mTFA, mLDFA, MPTA, and JLCA using the Shapiro–Wilk test and performed group-wise comparisons of their means and standard deviations (SDs) using repeated measures analysis of variance (ANOVA) between three groups or paired t-tests between two groups.

We evaluated the intraobserver and interobserver agreement of mTFA, mLDFA, MPTA, and JLCA between the readers and algorithm using the intraclass correlation coefficient (ICC) to assess measurement reproducibility. Altman considered an ICC of 0.81–1 as very good, 0.61–0.8 as good, and 0.41–0.6 as moderate (13). In the interobserver agreement test, we used the result of the second session for comparison when a reader performed two measurements.

Statistical significance was set at *p* < 0.05. We performed all statistical analyses using Medcalc software (version 20.114; MedCalc Software Ltd., Ostend, Belgium).

## Results

### Segmentation performance

As indicated in Table 1, we assessed the segmentation performance using metrics including global accuracy, mean accuracy, mean IoU, weighted IoU, and DSC to thoroughly analyze the results obtained in segmentation problems. The segmentation algorithm demonstrated excellent agreement with the human-annotated segmentation for all the anatomical regions, with an average DSC of 93% for the femoral head, 95% for the distal femur, 95% for the proximal tibia, 89% for the distal tibia, and 97% for the talus. Other values ranged from 96 to 98% for the femoral head, 95% to 96% for the distal femur, 96% to 98% for the proximal tibia, 93% to 96% for the distal tibia, and 94% to 98% for the talus.

**Table 1:** Segmentation accuracy measured using various evaluation metrics.

	Global accuracy	Mean accuracy	Mean IoU	Weighted IoU	Mean DSC
Femoral head	0.98	0.98	0.96	0.96	0.93
Distal femur	0.98	0.98	0.95	0.96	0.95
Proximal tibia	0.98	0.98	0.96	0.96	0.95
Distal tibia	0.98	0.98	0.93	0.96	0.89
Talus	0.98	0.98	0.94	0.96	0.97

*IoU* intersection over union, *DSC* dice similarity coefficients.

### Assessment of measurement comparisons to algorithms

**Figure 6:**
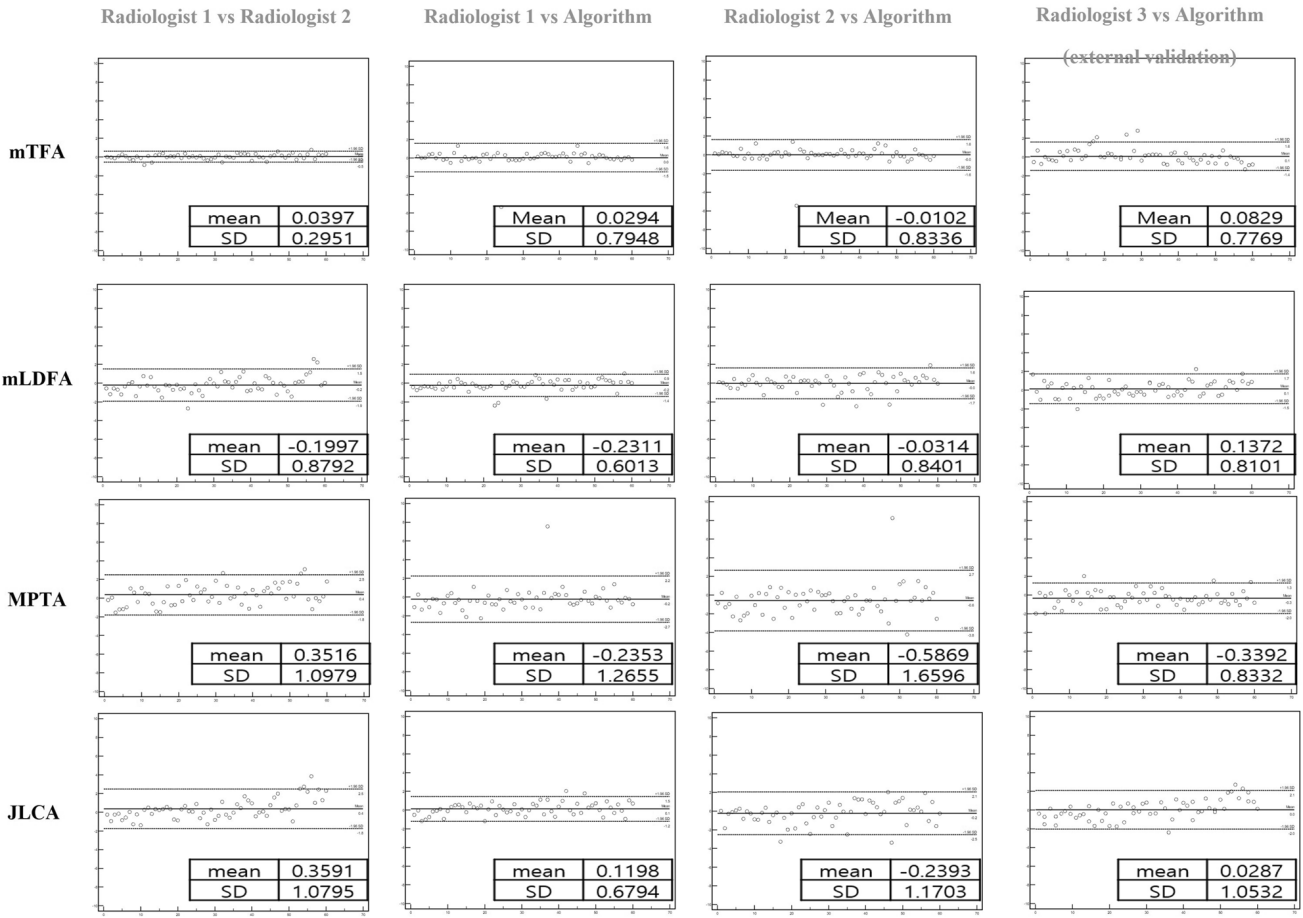
Comparative evaluation of reader and algorithm based on mechanical tibiofemoral angle (mTFA), mechanical lateral distal femoral angle (mLDFA), medial proximal tibial angle (MPTA), and joint line convergence angle (JLCA).

Measurements of the lower limb alignment did not significantly differ between the readers and algorithm in the internal institution test set, as shown in Table 2 (mTFA: Reader 1, 181.82° ± 3.39; Reader 2, 181.78° ± 3.33; Algorithm, 181.79° ± 3.48; mLDFA: Reader 1, 87.51° ± 1.96; Reader 2, 87.71° ± 1.8; Algorithm, 87.73° ± 1.86; MPTA: Reader 1, 86.76° ± 3.19; Reader 2, 86.41° ± 3.08; Algorithm, 86.99° ± 3.29; JLCA: Reader 1, 1.79° ± 1.43; Reader 2, 1.73° ± 1.07; Algorithm, 1.67° ± 1.41) (all *p* > 0.05). The average angle differences between the readers and algorithm in the internal and external institutions are shown in Fig. 6. The mean differences in mTFA, mLDFA, MPTA, and JLCA between the two readers were 0.04° ± 0.30, 0.20° ± 0.88, 0.35° ± 1.10, and 0.36° ± 1.08, respectively. The mean differences between Reader 1 and the algorithm and Reader 2 and the algorithm were 0.03° ± 0.79 and 0.01° ± 0.83 for mTFA, 0.23° ± 0.60 and 0.03° ± 0.84 for mLFDA, 0.23° ± 1.27 and 0.59° ± 1.66 for MPTA, and 0.12° ± 0.68 and 0.24° ± 1.17 for JLCA, respectively. based on a mechanical tibiofemoral angle. The intraobserver correlations (ICC range, 0.9836–0.9991) between sessions 1 and 2 for Reader 1 and the interobserver correlations (ICC range, 0.7751–0.9981) between Readers 1 and 2 were good to very good, as shown in Table 3. The ICC scores of angles measured by Reader 1, Reader 2, and the algorithm indicated good to very good agreement, as shown in Table 4 (ICC ranges: 0.9848–0.9865 for mTFA, 0.9443–0.9746 for mLDFA, 0.9273–0.9604 for MPTA, and 0.7213–0.9393 for JLCA).

**Table 2:** Details of manual and automatic measurements of lower limb alignment.

	Group	Mean	SD	P-value
mTFA	Reader1	181.82	3.39	0.998
	Reader 2	181.78	3.33	
	Algorithm	181.79	3.48	
mLDFA	Reader1	87.51	1.96	0.765
	Reader 2	87.71	1.8	
	Algorithm	87.73	1.86	
MPTA	Reader1	86.76	3.19	0.598
	Reader 2	86.41	3.08	
	Algorithm	86.99	3.29	
JLCA	Reader1	1.79	1.43	0.315
	Reader 2	1.43	1.07	
	Algorithm	1.67	1.41	

*SD* standard deviation, *mTFA* mechanical tibiofemoral angle, *mLDFA* mechanical lateral distal femoral articular angle, *MPTA* medial proximal tibial angle, *JLCA* joint line convergence angle.

**Table 3:** Details of intraobserver and interobserver agreement of lower limb alignment between readers.

	ICC		95% CI		P*-*value	
	R1 vs R1 (intraobserver)	R1 vs R2 (interobserver)	R1 vs R1 (intraobserver)	R1 vs R2 (interobserver)	R1 vs R1 (intraobserver)	R1 vs R2 (interobserver)
mTFA	0.9991	0.9981	0.9984–0.9995	0.9968–0.9988	< 0.0001	< 0.0001
mLDFA	0.9900	0.9420	0.9831–0.9940	0.9030–0.9654	< 0.0001	< 0.0001
MPTA	0.9949	0.9683	0.9914–0.9970	0.9470–0.9811	< 0.0001	< 0.0001
JLCA	0.9836	0.7751	0.9725–0.9902	0.6234–0.8656	< 0.0001	< 0.0001

*ICC* in-class correlation coefficient, *CI* confidence interval, *R1* reader 1, *R2* reader 2, *mTFA* mechanical tibiofemoral angle, *mLDFA* mechanical lateral distal femoral articular angle, *MPTA* medial proximal tibial angle, *JLCA* joint line convergence angle.

**Table 4:** Details of intraobserver and interobserver agreement of lower limb alignment between the readers and algorithm.

	ICC		95% CI		P-value	
	R1 vs Al	R2 vs Al	R1 vs Al	R2 vs Al	R1 vs Al	R2 vs Al
mTFA	0.9865	0.9848	0.9773–0.9919	0.9745–0.9909	< 0.0001	< 0.0001
mLDFA	0.9746	0.9443	0.9574–0.9848	0.9068–0.9668	< 0.0001	< 0.0001
MPTA	0.9604	0.9273	0.9336–0.9763	0.8782–0.9566	< 0.0001	< 0.0001
JLCA	0.9393	0.7213	0.8984–0.9638	0.5333–0.8335	< 0.0001	< 0.0001

*ICC* in-class correlation coefficient, *CI* confidence interval, *R1* reader 1, *R2* reader 2, *mTFA* mechanical tibiofemoral angle, *mLDFA* mechanical lateral distal femoral articular angle, *MPTA* medial proximal tibial angle, *JLCA* joint line convergence angle, *R1* reader 1, *R2* reader 2, *AI* artificial intelligence.

### Measurement times

The time taken for the manual measurements of lower limb alignment from the internal institution test set (n = 30) by the two readers averaged 86 min (average of 172 s/patient). In contrast, the time taken for computer-aided automatic measurements was 25 min, including the loading time for training data (average of 50 s/patient), which was 3.44 times faster than that for manual measurement. The processing time taken after data loading averaged 20 s/patient.

### External validation of the algorithm

External validation included 30 long-leg radiographs from consecutive patients at an external hospital. Intraobserver correlations (ICC ranges: 0.9393–0.9979) between sessions 1 and 2 for Reader 3 and the interobserver correlations (ICC ranges, 0.7126–0.9695) between the manual and automatic measurements were good to very good, as shown in Table 5. There was no statistically significant difference between the measurements of the lower limb alignment by the reader and algorithm in the external validation, as shown in Table 6 (mTFA: Reader 3, 181.37° ± 2.26; Algorithm, 181.26° ± 2.56; mLDFA: Reader 3, 86.92° ± 2.03; Algorithm, 86.80° ± 2.01; MPTA: Reader, 86.20° ± 1.65; Algorithm, 86.55° ± 1.66; JLCA: Reader 3, 0.40° ± 1.74; Algorithm 0.49° ± 1.58) (all *p* > 0.05). The average angle differences between the reader and algorithm are shown in Fig. 6.

**Table 5:** Details of intraobserver and interobserver agreement of lower limb alignment between the manual and automatic measurement on external validation.

	ICC		95% CI		P*-*value	
	R3 vs R3 (intraobserver)	R3 vs AI (interobserver)	R3 vs R3 (intraobserver)	R3 vs AI (interobserver)	R3 vs R3 (intraobserver)	R3 vs AI (interobserver)
mTFA	0.9979	0.9695	0.9965–0.9988	0.9489–0.9818	< 0.0001	< 0.0001
mLDFA	0.9830	0.9218	0.9716–0.9899	0.8692–0.9533	< 0.0001	< 0.0001
MPTA	0.9748	0.9199	0.9579–0.9850	0.8658–0.9521	< 0.0001	< 0.0001
JLCA	0.9353	0.7126	0.8916–0.9613	0.5189–0.8283	< 0.0001	< 0.0001

*ICC* in-class correlation coefficient, *CI* confidence interval, *R3* reader 3, *mTFA* mechanical tibiofemoral angle, *mLDFA* mechanical lateral distal femoral articular angle, *MPTA* medial proximal tibial angle, *JLCA* joint line convergence angle, *AI* artificial intelligence.

**Table 6:** Details of manual and automatic measurement of lower limb alignment on external validation.

	Group	Mean	SD	P-value
mTFA	Reader 3	181.37	2.26	0.3218
	Algorithm	181.26	2.56	
mLDFA	Reader 3	86.92	2.03	0.3975
	Algorithm	86.80	2.01	
MPTA	Reader 3	86.20	1.65	0.2492
	Algorithm	86.55	1.66	
JLCA	Reader 3	1.32	1.20	0.5024
	Algorithm	1.26	1.06	

*SD* standard deviation, *mTFA* mechanical tibiofemoral angle, *mLDFA* mechanical lateral distal femoral articular angle, *MPTA* medial proximal tibial angle, *JLCA* joint line convergence angle.

## Discussion

The variability of conventional alignment measurement causes controversy. Surgeons have reported inconsistencies and discordance between conventional radiographic measurements and intraoperative navigation measurements^16,17^. Wright et al. reported three sources of measurement inconsistency: physiological variations, procedure variability (inconsistent positioning), and intra- and interobserver variability^18^. The mean interobserver difference was 1.4° (SD = 1.1), and the mean intra-observer difference was 0.7° (SD = 0.9). Laskin et al. reported up to 7° variability in tibiofemoral angle measurements among 50 surgeons^19^. Automated measurement reduces these errors by minimising subjectivity.

We proposed a time-efficient system that automatically measures mTFA, mLDFA, MPTA, and JLCA from full-length leg weight-bearing radiographs. The system strongly correlated with the manual measurements in the internal and external institution tests.

Accurate segmentation is required for the automatic measurement of lower limb alignment. Previous studies performed femoral and tibial segmentation using a traditional spectral clustering and active shape model^20^ or unsupervised or atlas-guided approaches^21–23^. Deep-learning methods have been applied in image segmentation, with UNet being popular in the medical field. However, UNet may not be the most efficient option for relatively simple data (images with fewer large objects) as it may require more resources. In this study, a SegNet model was used for image segmentation.

There have been studies utilizing long leg radiographs to investigate detailed angular values related to coronal alignment^24–28^. However, these papers commonly employ a method where landmarks are directly annotated by humans, and algorithms are subsequently trained based on this annotated data. This approach inherently introduces a potential bias to the reference values, as the ground truth is produced by humans marking points manually. In contrast, our approach involves segmentation followed by the identification of landmarks using a predetermined rule-based system. This method has the potential to reduce interobserver agreement on ground truth, as it eliminates the reliance on manual point annotation by humans. Moreover, the segmentation mask generated by the algorithm can be used to identify new geometric landmarks.

Zheng et al. proposed a method for automatically measuring leg length discrepancy in a pediatric population using deep learning^29^. The method demonstrated a high concordance rate between manual and automatic segmentation of the pediatric leg, with a Dice value of 0.94. However, their study employed a wide exclusion criteria. In contrast, Schock et al. achieved a high level of concordance rate across a wide range of clinical and pathologic indications, with an average Sørensen–Dice coefficient of 0.97 for the femur and 0.96 for the tibia^10^.

In our internal validation, the readers and algorithm demonstrated a high concordance rate. The algorithm required 1 min/patient, in contrast to the manual measurement time of up to 3 min. In the external validation, the algorithm results significantly correlated with the manual measurements. However, the validation population consisted of young soldiers aged 20–30 years from a military hospital and may not represent the general population. JLCA values tended to be lower in military hospital patients than in those from the other included hospital. Nevertheless, these findings suggest that our algorithm may be useful in other populations.

Our study had several limitations. First, the training data did not include images from patients with skeletal dysplasia or hardware, limiting the clinical variability of the images. Second, several cases showed a large absolute error (> 5°) between manual and automated measurement results. Future studies should include a wider variety and number of training data to reduce these errors. Third, our study included a total of 374 images from 374 patients for algorithm development, which may be considered too few compared to those in larger studies. However, studies by Zheng et al. and Schock et al. enrolled 179 and 255 patients, respectively, and showed convincing results in their analyses, indicating that the number of cases analysed in our study (n = 374) was sufficient to demonstrate excellent performance^10,29^.

In conclusion, our deep-learning-based automated measurement algorithm accurately quantified the clinical values of lower limb alignment from long-leg radiographs and was faster than manual measurement was. The algorithm may be applied in clinical settings since it was validated for various patient images and clinical and pathological situations.

## Data Availability

As per Yonsei University Medical Center's data policy, which governs this research's location, authorization from both the "Data Asset Review Committee" and the "Data Review Board" is mandatory prior to exporting or revealing data. Consequently, adherence to Yonsei Medical Center's administrative protocols is essential to furnish data to an external researcher or institution. It's worth noting that this approval protocol is waived for internal researchers' studies, hence not being a requirement for the ongoing research. Currently, we are unable to provide the data; nevertheless, upon request, we can supply it once the aforementioned procedures are finalized. Contact: Sungjun Kim, AGN70@yuhs.ac.

## Code availability

The source code utilized in this research is publicly available for unrestricted access and use by anyone via the following link: 10.5281/zenodo.10838061. We encourage fellow researchers and practitioners to explore and utilize the codebase for their own purposes.

## References

[1] Burghardt RD, Hinterwimmer S, Burklein D, Baumgart R (2013). Lower limb alignment in the frontal plane: Analysis from long standing radiographs and computer tomography scout views: An experimental study. Arch. Orthop. Trauma Surg..

[2] Zampogna B (2015). Assessing lower limb alignment: Comparison of standard knee Xray vs long leg view. Iowa Orthop. J..

[3] Sharma L (2001). The role of knee alignment in disease progression and functional decline in knee osteoarthritis. JAMA.

[4] Felson DT (2013). Valgus malalignment is a risk factor for lateral knee osteoarthritis incidence and progression: Findings from the Multicenter osteoarthritis study and the osteoarthritis initiative. Arthritis Rheum..

[5] Sharma L (2013). The role of varus and valgus alignment in the initial development of knee cartilage damage by MRI: The MOST study. Ann. Rheum. Dis..

[6] Micicoi G (2021). Neutral alignment resulting from tibial vara and opposite femoral valgus is the main morphologic pattern in healthy middle-aged patients: An exploration of a 3D-CT database. Knee Surg. Sports Traumatol. Arthrosc..

[7] Thienpont E, Schwab PE, Cornu O, Bellemans J, Victor J (2017). Bone morphotypes of the varus and valgus knee. Arch. Orthop. Trauma Surg..

[8] Kijowski R, Liu F, Caliva F, Pedoia V (2020). Deep learning for lesion detection, progression, and prediction of musculoskeletal disease. J. Magn. Reson. Imaging.

[9] Gyftopoulos S (2019). Artificial intelligence in musculoskeletal imaging: current status and future directions. AJR Am. J. Roentgenol..

[10] Schock J (2021). Automated analysis of alignment in long-leg radiographs by using a fully automated support system based on artificial intelligence. Radiol. Artif. Intell..

[11] Badrinarayanan V, Kendall A, Cipolla R (2017). SegNet: A deep convolutional encoder-decoder architecture for image segmentation. IEEE Trans. Pattern Anal. Mach. Intell.

[12] Barber CB, Dobkin DP, Huhdanpaa H (1996). The quickhull algorithm for convex hulls. ACM Trans. Math. Softw. (TOMS).

[13] O'Rourke J (1985). Finding minimal enclosing boxes. Int. J. Comput. Inf. Sci..

[14] Zou KH, Wells WM, Kikinis R, Warfield SK (2004). Three validation metrics for automated probabilistic image segmentation of brain tumours. Stat. Med..

[15] Dice LR (1945). Measures of the amount of ecologic association between species. Ecology.

[16] Yaffe MA, Koo SS, Stulberg SD (2008). Radiographic and navigation measurements of TKA limb alignment do not correlate. Clin. Orthopaed. Relat. Res..

[17] Han SB, Kim HJ, Lee DH (2017). Effect of computer navigation on accuracy and reliability of limb alignment correction following open-wedge high tibial osteotomy: A meta-analysis. Biomed. Res. Int..

[18] Wright JG, Treble N, Feinstein AR (1991). Measurement of lower limb alignment using long radiographs. J. Bone Joint Surg. Br..

[19] Laskin, R. S. Alignment of total knee components, Vol. 7 62–72 (SLACK Incorporated Thorofare, NJ, 1984). 10.3928/0147-7447-19840101-09 24824049

[20] Wu, J. & Mahfouz, M. R. Robust x-ray image segmentation by spectral clustering and active shape model. *J. Med. Imaging (Bellingham)* **3**, 034005 (2016). 10.1117/1.JMI.3.3.034005 PMC5028420 27660806

[21] Gandhamal A, Talbar S, Gajre S, Hani A, Kumar D (2017). Automatic and unsupervised femur and tibia segmentation using magnetic resonance images. Osteoarth. Cartil..

[22] Gandhamal A (2017). Fully automated subchondral bone segmentation from knee MR images: Data from the osteoarthritis initiative. Comput. Biol. Med..

[23] Aprovitola A, Gallo L (2016). Knee bone segmentation from MRI: A classification and literature review. Biocybern. Biomed. Eng..

[24] Kim SE, Nam JW, Kim JI, Kim J-K, Ro DH (2024). Enhanced deep learning model enables accurate alignment measurement across diverse institutional imaging protocols. Knee Surg. Relat. Res..

[25] Jo C (2023). Deep learning-based landmark recognition and angle measurement of full-leg plain radiographs can be adopted to assess lower extremity alignment. Knee Surg. Sports Traumatol. Arthrosc..

[26] Nam HS (2023). Key-point detection algorithm of deep learning can predict lower limb alignment with simple knee radiographs. J. Clin. Med..

[27] Simon S (2022). Fully automated deep learning for knee alignment assessment in lower extremity radiographs: A cross-sectional diagnostic study. Skeletal Radiol..

[28] Meng X (2022). Fully automated measurement on coronal alignment of lower limbs using deep convolutional neural networks on radiographic images. BMC Musc. Disord..

[29] Zheng Q, Shellikeri S, Huang H, Hwang M, Sze RW (2020). Deep learning measurement of leg length discrepancy in children based on radiographs. Radiology.

